# Native PLGA nanoparticles attenuate Aβ-seed induced tau aggregation under in vitro conditions: potential implication in Alzheimer’s disease pathology

**DOI:** 10.1038/s41598-023-50465-x

**Published:** 2024-01-02

**Authors:** Pallabi Sil Paul, Tark Patel, Jae-Young Cho, Allan Yarahmady, Aria Khalili, Valentyna Semenchenko, Holger Wille, Marianna Kulka, Sue-Ann Mok, Satyabrata Kar

**Affiliations:** 1 Departments of Medicine (Neurology), Centre for Prions and Protein Folding Diseases, University of Alberta, Edmonton, AB T6G 2M8 Canada; 2 Departments of Biochemistry, Centre for Prions and Protein Folding Diseases, University of Alberta, Edmonton, AB T6G 2M8 Canada; 3 Nanotechnology Research Centre, National Research Council Canada, Edmonton, AB T6G 2M9 Canada; 4 Department of Medical Microbiology and Immunology, University of Alberta, Edmonton, AB T6G 2E1 Canada; 5 Centre for Prions and Protein Folding Diseases, Departments of Medicine (Neurology) and Psychiatry, University of Alberta, Edmonton, AB T6G 2M8 Canada

**Keywords:** Neuroscience, Diseases, Neurology, Nanoscience and technology

## Abstract

Evidence suggests that beta-amyloid (Aβ)-induced phosphorylation/aggregation of tau protein plays a critical role in the degeneration of neurons and development of Alzheimer’s disease (AD), the most common cause of dementia affecting the elderly population. Many studies have pursued a variety of small molecules, including nanoparticles conjugated with drugs to interfere with Aβ and/or tau aggregation/toxicity as an effective strategy for AD treatment. We reported earlier that FDA approved PLGA nanoparticles without any drug can attenuate Aβ aggregation/toxicity in cellular/animal models of AD. In this study, we evaluated the effects of native PLGA on Aβ seed-induced aggregation of tau protein using a variety of biophysical, structural and spectroscopic approaches. Our results show that Aβ_1-42_ seeds enhanced aggregation of tau protein in the presence and absence of heparin and the effect was attenuated by native PLGA nanoparticles. Interestingly, PLGA inhibited aggregation of both 4R and 3R tau isoforms involved in the formation of neurofibrillary tangles in AD brains. Furthermore, Aβ seed-induced tau aggregation in the presence of arachidonic acid was suppressed by native PLGA. Collectively, our results suggest that native PLGA nanoparticles can inhibit the Aβ seed-induced aggregation of different tau protein isoforms highlighting their therapeutic implication in the treatment of AD.

## Introduction

Alzheimer’s disease (AD), the most prevalent cause of dementia in the elderly, is characterized by the progressive accumulation of extracellular neuritic plaques, intracellular neurofibrillary tangles (NFTs) and the loss of neurons in discrete regions of the brain. Structurally, neuritic plaques contain a central deposit of aggregated β-amyloid (Aβ) peptides, whereas NFTs comprise of paired helical filaments (PHFs) formed by hyperphosphorylation of microtubule-associated tau protein^1,2^. Although Aβ deposition presages tau pathology, the number of NFTs in the brain, unlike neuritic plaques, correlates with clinical severity of dementia and neurodegeneration in AD^2,3^. Tau is a soluble cytoplasmic protein comprising a N-terminal domain, proline-rich domain, microtubule binding domain (MTBD) and C-terminal domain. Tau is primarily evident in neurons and adult human neurons express six different tau isoforms ranging from 352 to 441 amino acids containing 0, 1 or 2 N-terminal repeats (0N/1N/2N) with 3- or 4-MTBD repeats (4R/3R)^4^. Under normal conditions tau binds and stabilizes microtubules via site-specific phosphorylation-dephosphorylation process mediated by kinases and phosphatases, respectively. However, hyperphosphorylation of tau in pathological conditions promotes their detachment from microtubules leading to fibrillization into single-straight filaments/PHFs and deposition as NFTs in AD brains^5–8^. Conversion of tau from soluble monomer into oligomers/aggregates plays an important role not only in the loss of neurons but also in the “seed-induced” regional spreading of disease pathology^9,10^. Thus, preventing tau aggregation has long been deemed as a promising strategy for delaying the onset and/or progression of AD.

Accompanying tau, various isoforms of Aβ peptides containing 39–43 amino acids are generated from amyloid precursor protein (APP), but the two most prevalent isoforms found in the normal brain are Aβ_1-40_ and Aβ_1-42_. Although Aβ_1-42_ constitutes ~ 10% of the total Aβ secreted from cells, it aggregates faster and found to be more toxic to neurons than Aβ_1-40._ A progressive increase in Aβ levels, a consequence of enhanced production and/or decreased clearance, is believed to underlie the conversion of Aβ from their soluble monomers into aggregated states leading to degeneration of neurons and development of AD^5,11^. Mounting evidence gathered over the last two decades indicates that synergistic interactions between Aβ and tau, rather than effects of the individual protein may underlie the cause and progression of AD pathology^3^. This is supported by data showing that (i) Aβ accelerates tau phosphorylation and aggregation, (ii) complexes of Aβ bound to tau are detected in AD brain extracts, (iii) Aβ core region can bind directly to tau and (iv) Aβ aggregates can trigger/propagate tau pathology in cultured cells and animal models^12–15^. Notwithstanding the appearance of Aβ aggregates prior to tau pathology, the entwined nature of these two proteins necessitates an AD treatment strategy targeting simultaneously both proteins.

Over the last decade, a number of small molecules including nanoparticles, which are less than 100 nm in diameter with unique ability to transport drugs/agents across the blood brain-barrier (BBB), have been studied as novel therapeutic modalities to arrest/delay AD pathology^16,17^. In fact, a multitude of polymorphic nanoparticles when functionalized with molecules/agents such as curcumin, methylene blue, resveratrol, quercetin and polyphenols have been shown to attenuate Aβ/tau aggregation and toxicity in cellular or animal models of AD by improving the BBB permeability or binding affinity^18–21^. Surface charge and hydrophobicity enable the conjugated nanoparticles to bind Aβ or tau leading to inhibition of aggregation or dissociation of preformed fibrils^22^. Interestingly, acidic poly (D,L-lactide-co-glycolide) (PLGA) nanoparticles, which are synthesized from glycolic acid and lactic acid, have long been used as delivery vehicles for a variety of drugs, proteins and other macromolecules. Indeed, PLGA-encapsulated drugs/agents such as donepezil, memantine, curcumin, quercetin, rivastigmine and selegiline etc. exhibit beneficial effects on cellular and/or animal models of AD^23–26^. Apart from functionalized PLGA, we recently reported that PLGA nanoparticles without any drug/agent (i.e. native PLGA) can suppress Aβ aggregation/toxicity in cellular and animal models of AD^27–29^. However, no information is currently available if native PLGA can influence the aggregation, seeding or spreading of tau protein that acts synergistically with Aβ to trigger AD pathogenesis. In this study, we revealed that native PLGA nanoparticles can inhibit Aβ seed-induced aggregation of tau protein highlighting its therapeutic potential in regulating tau pathology in AD brains.

## Results

### In vitro aggregation of tau protein

**Figure 1:**
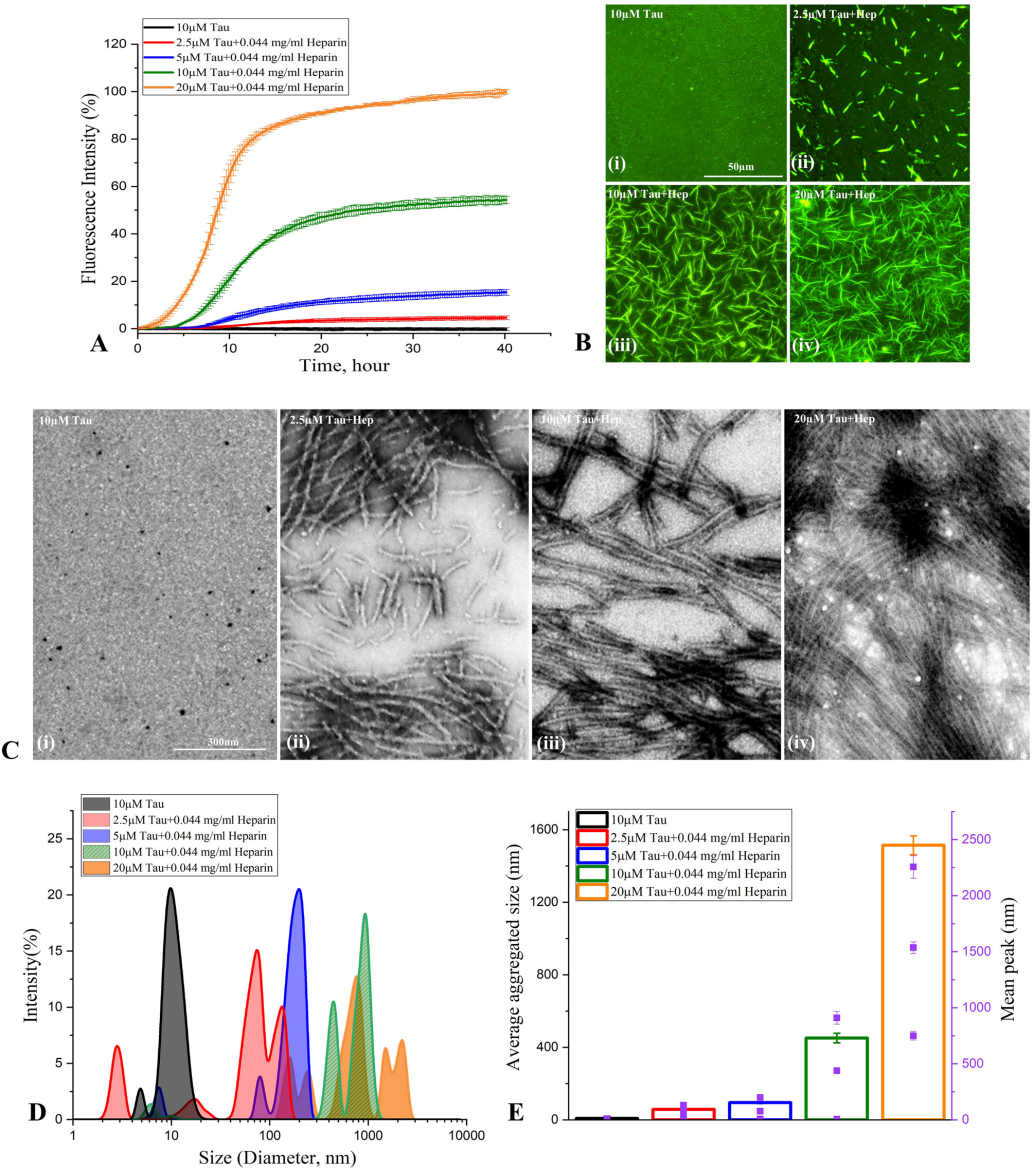
Tau aggregation in the presence and absence of heparin. (**A**–**C**) ThT kinetic assays showing the spontaneous aggregation curves (**A**) and the corresponding fluorescence (**B**) as well as STEM (**C**) images of 0N4R tau in the absence and presence of heparin over 40 h incubation. Note the aggregation of 0N4R tau increases dose-dependently in presence of heparin which reaches plateaus over time as indicated by ThT fluorescence levels. Tau, as expected, did not aggregate in the absence of heparin (**A**). All ThT kinetic graphs represented average mean ± SEM of three separate experiments, each performed with six replicates for each condition. The fluorescence (**B**) and STEM (**C**) images reflecting tau aggregation at different concentrations were taken following 40 h incubation. (**D**,**E**) DLS analysis showing differential increase in the diameter of aggregated tau as a function of concentrations in the presence and absence of heparin following 40 h incubation as depicted by size distribution curves (**D**) and histogram (**E**) representing average aggregate size (left side Y axis) and mean peaks of tau aggregates for a given condition (right side Y axis).

Tau fibrils formed by repetitive cross-beta sheets stabilized via different molecular interactions play a critical role in AD pathogenesis^30^. Detection of tau fibrils/aggregates is usually carried out using Thioflavin T (ThT), a classic amyloid dye which exhibits strong fluorescence emission upon binding to cross-β fibril structures^31^. To determine how Aβ_1-42_ seed can influence the fibrillization of tau protein, we first evaluated aggregation kinetics of human 0N4R tau with or without heparin using the ThT assay at 37 °C for a period of 40 h. As reported earlier^4–6^, tau did not aggregate in the absence of heparin but showed aggregation with increasing concentrations in presence of heparin (Fig. 1A). The tau aggregation kinetics in the presence of heparin displayed a sigmoidal curve with a lag phase and elongation phase followed by saturation phase. As the concentration of tau increased from 2.5 to 20 µM, the lag phase decreased but aggregation rate and amount of fibril formation increased with time and then reached saturation by 15 h of incubation (Fig. 1A, Supplementary Fig. 1A,B). The propensity of tau aggregation at saturation was supported by our fluorescence imaging after ThT labelling (Fig. 1B) as well as Scanning transmission electron microscopy (STEM) images (Fig. 1C) revealing a dose-dependent increased number of large fibrils at 40 h of incubation. The conversion of tau from their monomeric to fibrillar state assessed by dynamic light scattering (DLS) also showed alteration in tau hydrodynamic radius from ~ 10–100 nm to ~ 100–2500 nm over 40 h incubation, signifying the formation of higher-ordered entities with increasing concentrations (Fig. 1D,E). It is evident from our results that enhanced aggregation of tau occurs as a function of time over 40 h incubation (see Supplementary Fig. 1C,D).

### Effect of Aβ_1-42_-seed on tau aggregation

**Figure 2:**
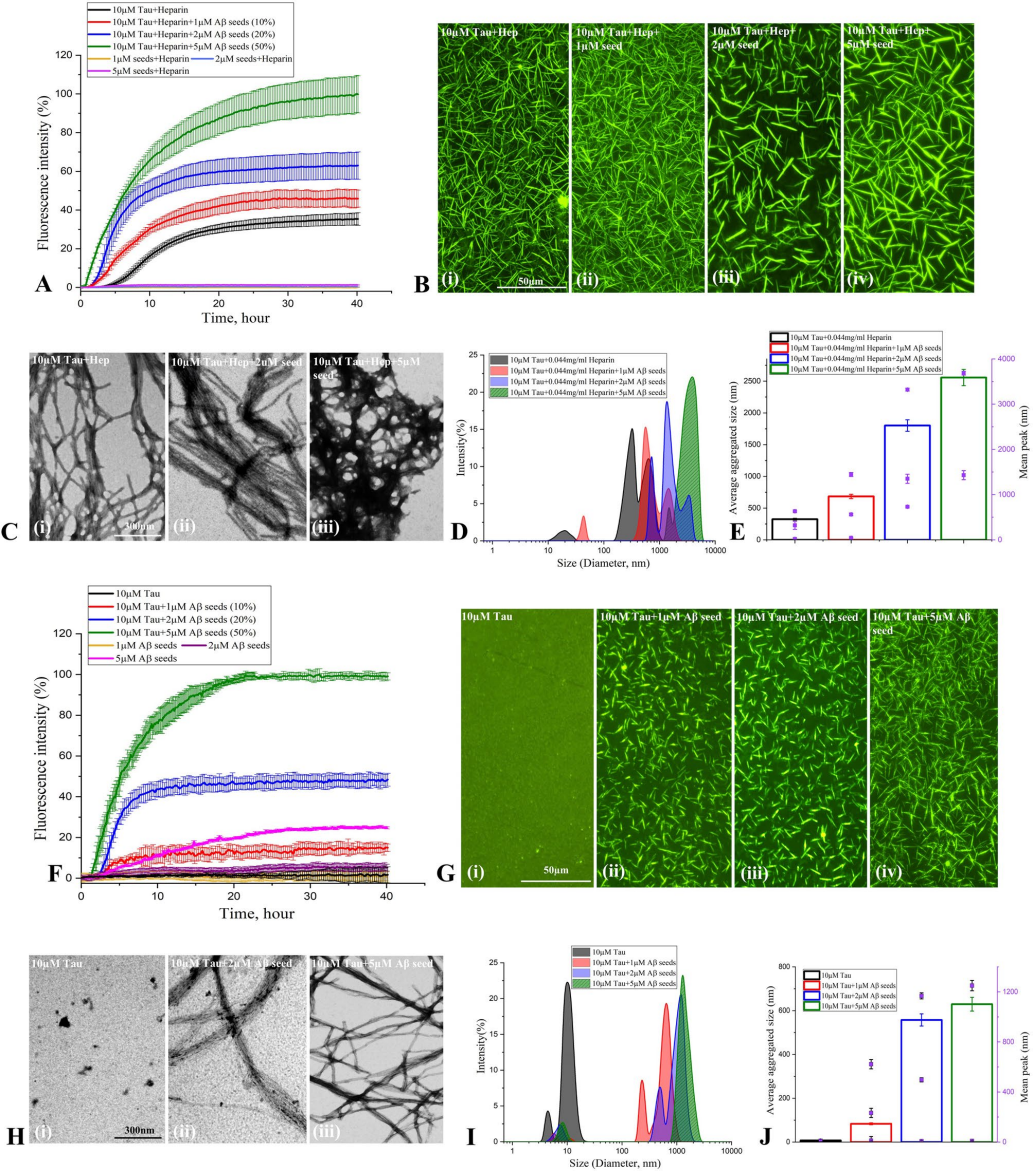
Aβ_1-42_ seed-induced tau aggregation. (**A**–**C**) ThT kinetic assays showing aggregation curves (**A**) and the corresponding fluorescence (**B**) as well as STEM (**C**) images of 10 µM 0N4R tau in the presence of heparin with or without 1–5 µM Aβ_1-42_ seeds. Note the aggregation of 10 µM 0N4R tau increases as a function of Aβ_1-42_ seed concentrations which reaches plateaus over time as indicated by ThT fluorescence levels (**A**). The fluorescence (**B**) and STEM (**C**) images reflecting increase tau aggregation with increasing concentrations of Aβ_1-42_ seed were taken following 40 h incubation. (**D**,**E**) DLS analysis showing differential increase in the diameter of aggregated tau as a function of Aβ_1-42_ seed concentrations in the presence of heparin following 40 h incubation as depicted by size distribution curves (**D**) and and histogram (**E**) representing average aggregate size (left side Y axis) and mean peaks of tau aggregates for a given condition (right side Y axis). (**F**–**J**) ThT kinetic assays showing the spontaneous aggregation curves (**F**) and the corresponding fluorescence (**G**) as well as STEM (**H**) images of 10 µM 0N4R tau in the absence of heparin with or without 1–5 µM Aβ_1-42_ seeds. Note the aggregation of 10 µM 0N4R tau increases as a function of Aβ_1-42_ seed concentrations which reaches plateaus over time as indicated by ThT fluorescence levels. Different concentrations of Aβ_1-42_ seed without tau is found to aggregate to a lesser extent (**F**). The fluorescence (**G**) and STEM (**H**) images reflecting tau aggregation with increasing concentrations of Aβ_1-42_ seed were taken following 40 h incubation. (**I**,**J**) DLS analysis showing differential increase in the diameter of aggregated tau as a function of Aβ_1-42_ seed concentrations in the absence of heparin following 40 h incubation as depicted by size distribution curves (**I**) and and histogram (**J**) representing average aggregate size (left side Y axis) and mean peaks of tau aggregates for a given condition (right side Y axis). All ThT kinetic graphs represented average mean ± SEM of three separate experiments, each performed with six replicates for each condition.

To investigate the effect of Aβ_1-42_ seed on tau aggregation, we performed ThT kinetics assays for 40 h at 37 °C by incubating 10 µM 0N4R tau with heparin in the presence or absence of freshly prepared 1–5 µM Aβ_1-42_ seeds. Our results clearly showed that increasing concentrations of Aβ_1-42_ seeds alone (without tau) did not exhibit profound altered aggregation profile over 40 h incubation (Supplementary Fig. 1E,F), but dose-dependently enhanced the rate of tau fibril formation as indicated by the decrease in lag phase values (Fig. 2A, Supplementary Fig. 1G,H). It is of interest to note that the aggregation profile of tau with 1 µM Aβ_1-42_ seed + heparin is higher than those observed with heparin alone (Fig. 2A). The augmented fibrilization of 0N4R tau protein in the presence of Aβ_1-42_ seed was confirmed by fluorescence (Fig. 2B) as well as STEM (Fig. 2C) imaging which depicted the morphology of tau fibrils. Our DLS analysis also showed a size increase from ~ 100–600 nm to ~ 1000–3000 nm in hydrodynamic radii of tau aggregates with rising concentration of Aβ_1-42_ seeds (Fig. 2D,E). To determine if Aβ_1-42_ seed can influence the aggregation of 0N4R tau in the absence of heparin, we performed ThT kinetic assays of 10 µM 0N4R tau with or without 1–5 µM Aβ_1-42_ seed for 40 h (Fig. 2F, Supplementary Fig. 1I,J). Our results revealed that Aβ_1-42_ seed dose-dependently enhanced the aggregation of tau protein compared to those observed in the absence of Aβ_1-42_ seed (Fig. 2F–J). It is also of interest to note that Aβ_1-42_ seeds and heparin appear to have a cumulative effect on tau aggregation compared to Aβ_1-42_ seeds or heparin alone as evident from the magnitude of fluorescence intensity in ThT kinetic assays (Supplementary Fig. 2A). The effects of Aβ_1-42_ seeds on tau aggregation were validated by fluorescence and STEM imaging (Fig. 2G,H) as well as DLS (Fig. 2I,J) analysis. Based on these results, our subsequent studies were carried out with 10 µM 0N4R tau in the presence of a 2 µM Aβ_1-42_ (i.e. 20% seed concentration).

### Effect of PLGA on Aβ_1-42_ seed-induced tau aggregation

**Figure 3:**
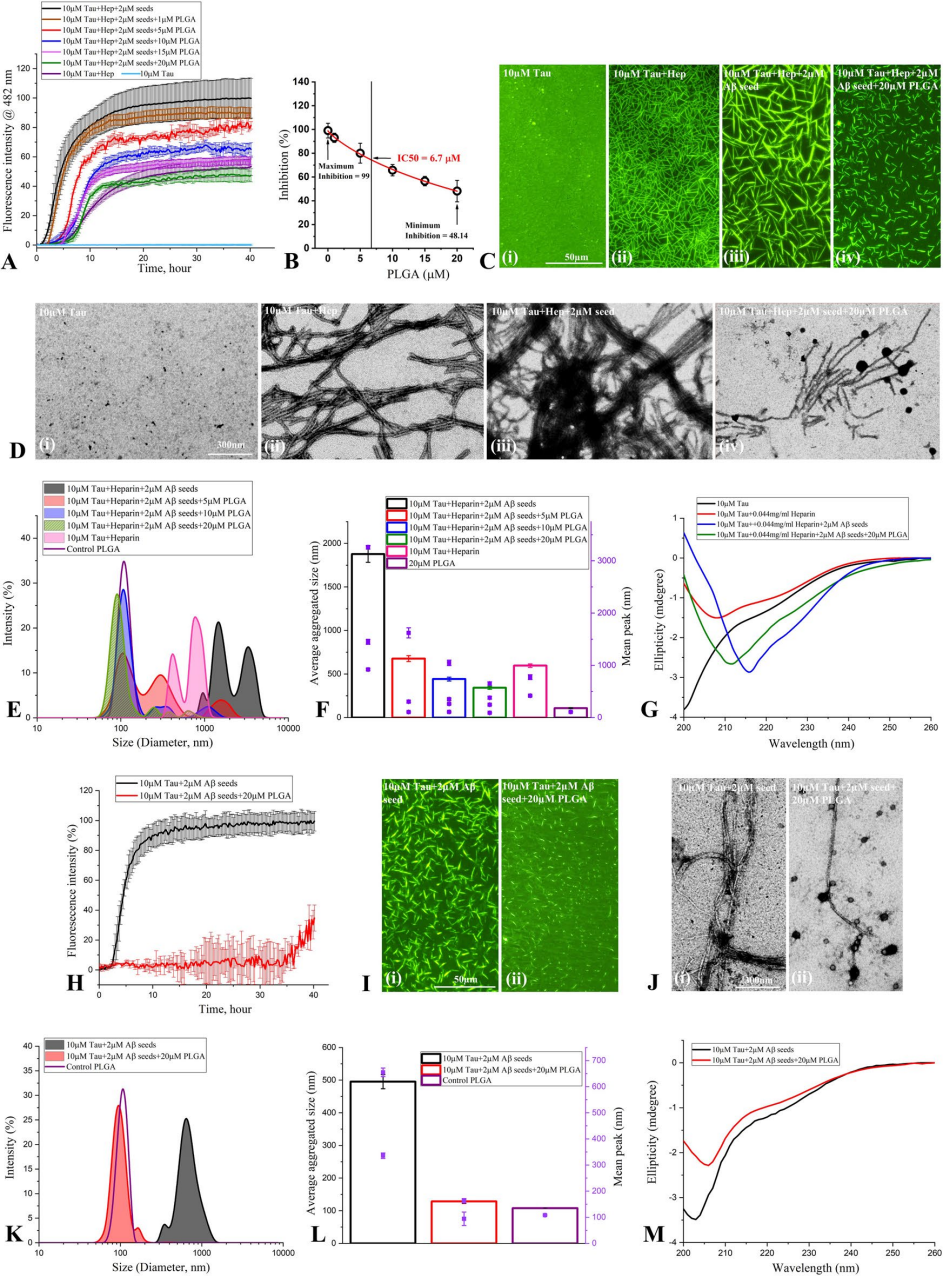
Attenuation of Aβ_1-42_ seed-induced tau aggregation by PLGA. (**A**–**D**) Native PLGA dose-dependently (1–20 µM) attenuates Aβ_1-42_ seed-induced spontaneous aggregation of 10 µM 0N4R tau in the presence of heparin as revealed by ThT kinetic assays (**A**,**B**) and respective fluorescence (**C**) as well as STEM (**D**) images over 40 h incubation. Note the IC50 value of native PLGA in inhibiting 10 µM 0N4R tau over 40 h incubation (**B**). The fluorescence (**C**) and STEM (**D**) images of PLGA untreated and treated tau samples in presence of heparin were taken after 40 h incubation. Note the relative decrease in the amount of tau fibrils in presence of different concentrations of PLGA. (**E–G**) DLS analysis (**E**,**F**) and CD spectra (**G**) of 10 µM 0N4R tau in the presence and absence of PLGA following 40 h incubation. DLS analysis reveals a differential decrease in the diameter of tau in the presence of different concentrations of PLGA compared to control tau as depicted by size distribution curves (**E**) and and histogram (**F**) representing average aggregate size (left side Y axis) and mean peaks of tau aggregates for a given condition (right side Y axis). CD spectra (**G**) showing β-sheet content following incubation of 10 µM 0N4R tau in the absence and presence of 20 µM PLGA after 40 h incubation. Note the decreased formation of β-sheet rich secondary structure in the presence of PLGA. (**H**–**M**) Native PLGA (20 µM) attenuates spontaneous aggregation of 10 µM 0N4R tau in the absence of heparin as revealed by ThT kinetic assays (**H**) and respective fluorescence (**I**) as well as STEM (**J**) images over 40 h incubation. The overall amount of tau aggregation are found to decrease in presence of 20 µM PLGA. The fluorescence (**I**) and STEM (**J**) images of PLGA untreated and treated tau samples in the absence of heparin were taken after 40 h incubation. Note the relative decrease in the amount of tau fibrils in the presence of PLGA. (**K**–**M**) DLS analysis (**K**,**L**) and CD spectra (**M**) of 10 µM 0N4R tau with or without 20 µM PLGA in the absence of heparin following 40 h incubation. DLS analysis reveals a differential decrease in the diameter of tau in the presence of 20 µM PLGA compared to control tau as depicted by size distribution curves (**K**) and and histogram (**L**) representing average aggregate size (left side Y axis) and mean peaks of tau aggregates for a given condition (right side Y axis). CD spectra (**M**) showing β-sheet content following incubation of 10 µM tau in the absence and presence of 20 µM PLGA after 40 h incubation. Note the decreased formation of β-sheet rich secondary in the presence of PLGA. All ThT kinetic graphs represented average mean ± SEM of three separate experiments, each performed with six replicates for each condition.

Prior to assessing the effects of native PLGA on tau aggregation, we depicted that PLGA nanoparticles displayed a diameter of ~ 100 nm with spheroidal morphology as observed with DLS and STEM analysis, respectively (Supplementary Fig. 2B,C). Subsequently, we determined the effects of 1–20 µM native PLGA on 2 µM Aβ_1-42_ seed-induced aggregation of 10 µM 0N4R tau with heparin using ThT kinetic assay. Our results indicate that PLGA dose-dependently inhibited Aβ seed-induced tau aggregation over a 40 h incubation period at 37 °C with an IC50 of 6.7 µM (Fig. 3A,B). At the low concentration of 1–5 µM, PLGA displayed only a modest inhibitory effect, whereas 10 µM PLGA noticeably attenuated tau aggregation compared to untreated samples. When the PLGA concentration was increased to 20 µM, not only a much stronger inhibition was observed, but the kinetic profile of tau aggregation was found to be somewhat similar to those observed without Aβ-seed (Fig. 3A). Increasing concentrations of PLGA were found to prolong the lag time as well as decrease the rate of Aβ-induced tau fibrilization (Supplementary Fig. 2D,E). Suppression of tau aggregation over 40 h incubation by 1, 5, 10, 15 and 20 µM native PLGA was found to be ~ 10.2%, ~ 21%, ~ 33.8%, ~ 42.7% and ~ 52.4%, respectively (Fig. 3A, Supplementary Fig. 2E). The inhibitory effect of PLGA on Aβ seed-induced tau aggregation at saturation was further substantiated by fluorescence imaging which revealed fewer tau fibrillar entities as a function of increasing PLGA concentrations (Fig. 3C).

To explore the structural details of tau aggregates in the absence and presence of 20 µM native PLGA after 40 h incubation, we used STEM to examine aggregate morphology at higher resolution. Our data showed that PLGA nanoparticles were directly associated with tau fibers and decreased their aggregation leading to the generation of a heterogeneous population of smaller tau aggregates (Fig. 3D). While Aβ seed-induced tau fibrils after 40 h incubation in the absence of PLGA appeared as long fibers, the PLGA-treated samples displayed mostly as short, twisted fibers and to a smaller extent as globular aggregates without any filamentous structure, indicating a possible shift/formation of shorter fibers (Fig. 3D). To confirm an overall decrease in the fibrillar size/entities of Aβ seed-induced tau fibers following PLGA treatment, we carried out DLS analysis of tau samples after performing aggregation kinetic assays. Our results, as expected, showed a decrease in hydrodynamic radii of tau aggregates with multiple peaks at increasing PLGA concentrations (Fig. 3E,F). To better understand the architectural changes responsible for the transformation of long tau fibrils into smaller aggregates after PLGA treatment, we performed Circular dichroism (CD) spectroscopy of Aβ seed-induced 10 µM tau samples incubated with or without 20 µM PLGA (Fig. 3G). Consistent with earlier reports^29^, our results indicated that tau samples after 40 h incubation without heparin or Aβ seed demonstrated a random coil structure (random coil and others 91.1%, α-helix 2.6%, β-sheet 6.3%) with minima around 200 nm, indicating an absence of aggregate formation. In the presence of Aβ seed and heparin, tau exhibited a change in its conformation from a random coil to a predominant β-sheet structure (random coil and others 16%, α-helix 4.8%, β-sheet 79.2%) displaying minima around 216 nm with a faint shoulder peak at 222 nm. In contrast to tau sample with Aβ seed and heparin, the presence of PLGA enhanced the α-helical and decreased β-sheet contents (random coil and others 9.3%, α-helix 25%, β-sheet 65.7%) observed at 216 nm (Fig. 3G).

**Figure 4:**
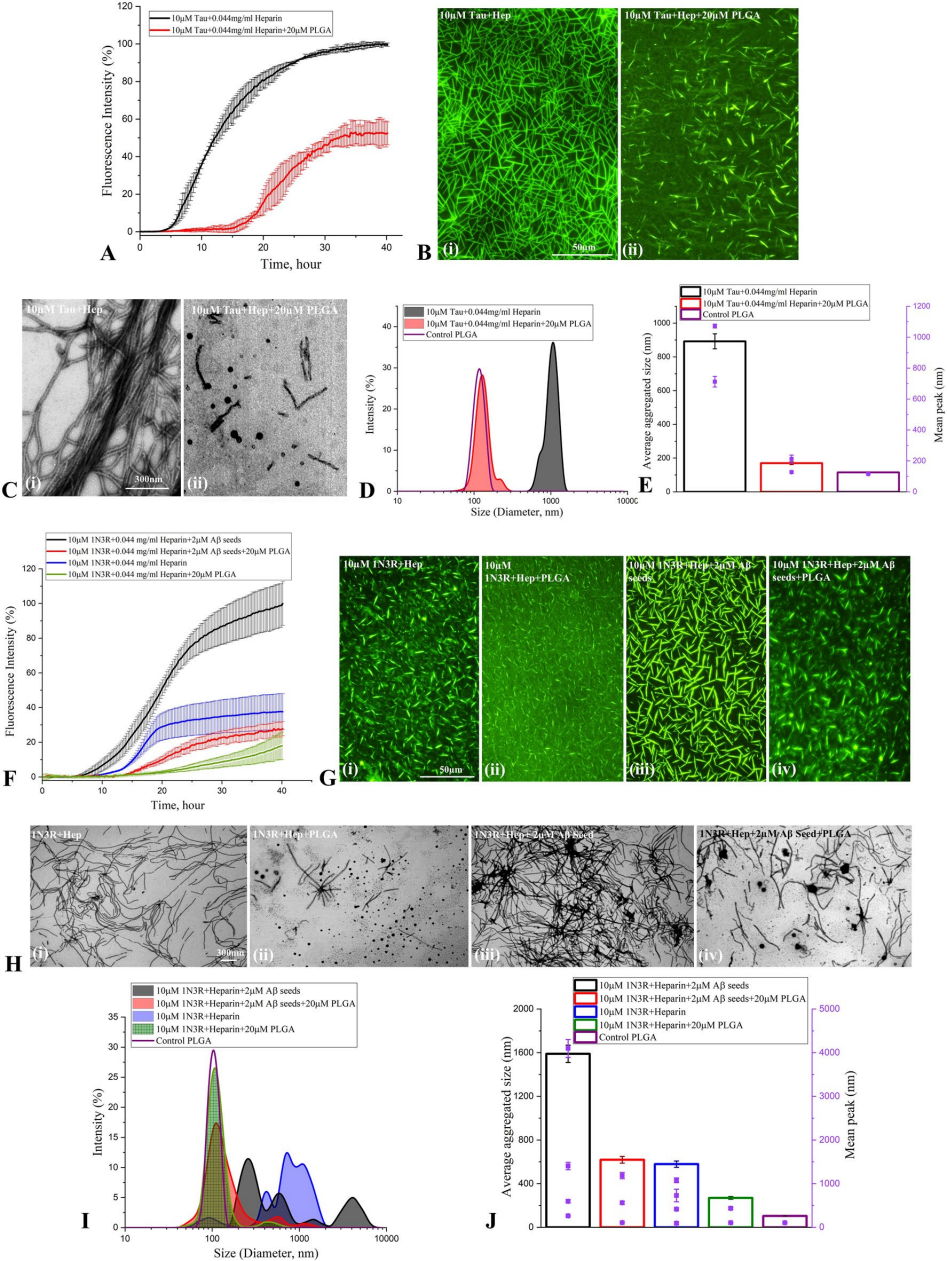
Attenuation of tau aggregation by PLGA. (**A**–**C**) PLGA (20 µM) attenuates aggregation of 10 µM 0N4R tau in presence of heparin without Aβ_1-42_ seed as revealed by ThT kinetic assays (**A**) and respective fluorescence (**B**) as well as STEM (**C**) images over 40 h incubation. The fluorescence (**B**) and STEM (**C**) images showed the relative decrease in the amount of tau fibrils in presence of 20 µM PLGA. (**D**,**E**) DLS analysis of 10 µM 0N4R tau + heparin ± PLGA without Aβ_1-42_ seeds revealed a differential decrease in the diameter of tau in the presence of PLGA compared to control tau as depicted by size distribution curves (**D**) and and histogram (**E**) representing average aggregate size (left side Y axis) and mean peaks of tau aggregates for a given condition (right side Y axis). (**F**–**J**) Native PLGA (20 µM) attenuates Aβ_1-42_ seed-induced aggregation of 10 µM 1N3R tau in the presence of heparin as revealed by ThT kinetic assays (**F**) and respective fluorescence (**G**) as well as STEM (**H**) images over 40 h incubation. The fluorescence (**G**) and STEM (H) images showed the relative decrease in the amount of tau fibrils in presence of 20 µM PLGA. (**I**,**J**) DLS analysis of Aβ_1-42_ seed-induced 10 µM 1N3R tau reveals a differential decrease in the diameter of tau in the presence of PLGA compared to control tau as depicted by size distribution curves (**I**) and and histogram (**J**) representing average aggregate size (left side Y axis) and mean peaks of tau aggregates for a given condition (right side Y axis). All ThT kinetic graphs represented average mean ± SEM of three separate experiments, each performed with six replicates for each condition.

**Figure 5:**
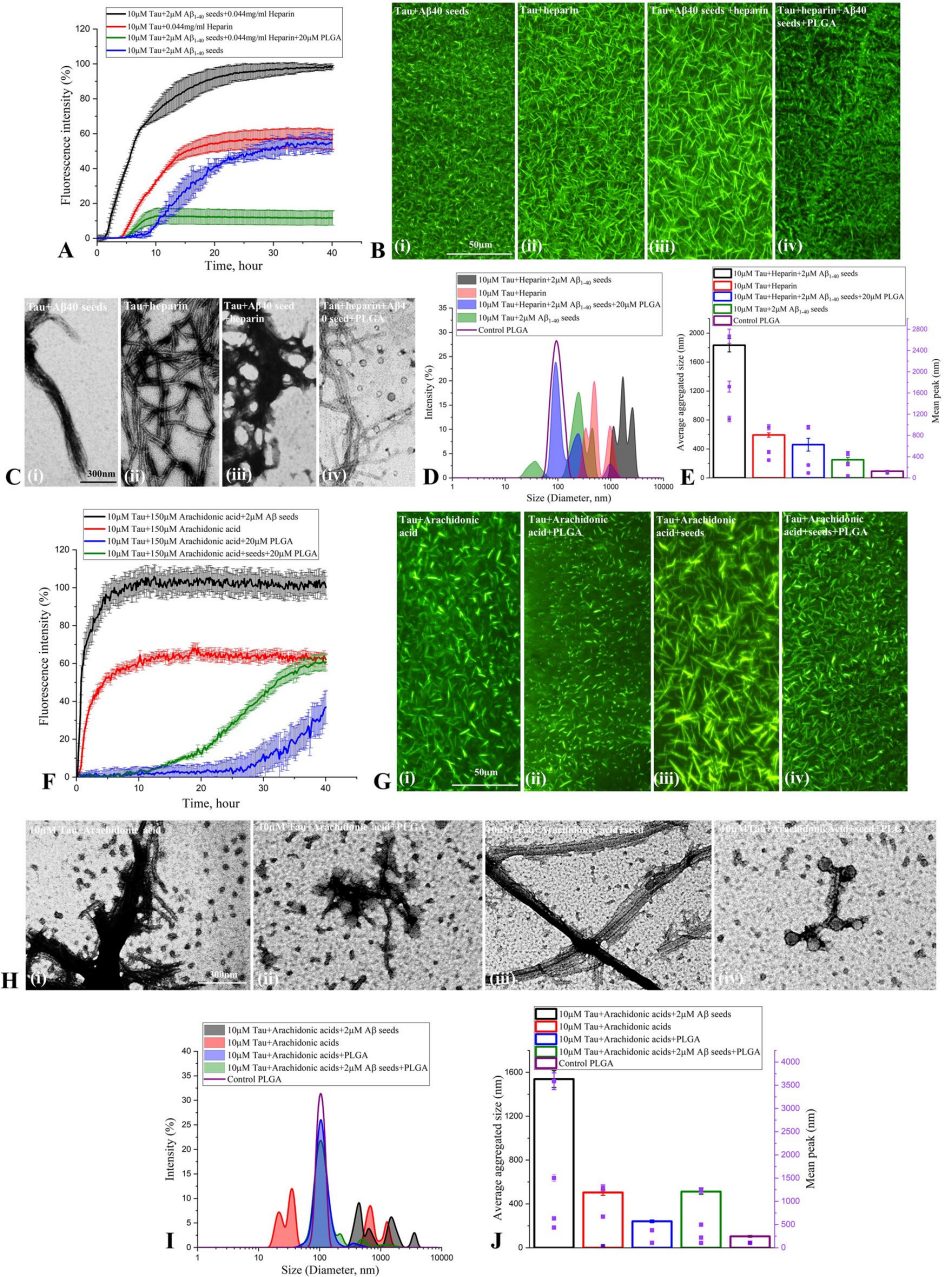
PLGA attenuates Aβ_1-40_ and arachidonic acid seed-induced tau aggregation. (**A**–**C**) Native PLGA (20 µM) attenuates Aβ_1-40_ seed-induced aggregation of 10 µM 0N4R tau in presence and absence of heparin as revealed by ThT kinetic assays (**A**) and respective fluorescence (**B**) as well as STEM (**C**) images over 40 h incubation. The fluorescence (**B**) and STEM (**C**) images showed the relative decrease in the amount of tau fibrils in presence of 20 µM PLGA. (**D**,**E**) DLS analysis of 10 µM 0N4R tau following 40 h incubation reveals a differential decrease in the diameter of tau in the presence of 20 µM PLGA compared to control tau as depicted by size distribution curves (**D**) and and histogram (**E**) representing average aggregate size (left side Y axis) and mean peak diameters of tau aggregates for a given condition (right side Y axis). (**F**–**H**) PLGA (20 µM) attenuated Aβ_1-42_ seed-induced aggregation of 10 µM 0N4R tau in presence of arachidonic acid as revealed by ThT kinetic assays (**F**) and respective fluorescence (**G**) as well as STEM (**H**) images over 40 h incubation. The fluorescence (**G**) and STEM (**H**) images showed the relative decrease in the amount of tau fibrils in the presence of PLGA. (**I**,**J**) DLS analysis of Aβ_1-42_ seed-induced 10 µM 0N4R tau reveals a differential decrease in the diameter of tau in the presence of PLGA compared to control tau as depicted by size distribution curves (**I**) and and histogram (**J**) representing average aggregate size (left side Y axis) and mean peaks of tau aggregates for a given condition (right side Y axis). All ThT kinetic graphs represented average mean ± SEM of three separate experiments, each performed with six replicates for each condition.

To establish if PLGA can influence Aβ seed-induced tau aggregation in the absence of heparin, we evaluated the effects of 20 µM native PLGA on 2 µM Aβ_1-42_ seed-induced aggregation of 10 µM tau without heparin using ThT kinetic assay, fluorescence imaging, STEM, DLS and CD spectroscopy. Our data clearly reveal that native PLGA can suppress Aβ_1-42_ seed-induced tau aggregation (~ 65%) in the absence of heparin (Fig. 3H–L, Supplementary Fig. 3A,B). This is supported by CD spectroscopy results showing an increase in the α-helical content from 5.7 to 9.8% and a decrease in the β-sheet content from 23.5 to 19.8% in presence of PLGA, suggesting a decrease of the conformational transition of tau from α-helix to β-sheets (Fig. 3M). In parallel, we showed that 20 µM native PLGA can also decrease heparin-induced tau aggregation (~ 47%) in the absence of Aβ_1-42_ seed (Fig. 4A–E; Supplementary Fig. 3C,D). Subsequently, we demonstrated that Aβ_1-42_ seed-induced aggregation of 10 µM 1N3R tau can be attenuated by 20 µM PLGA in the presence of heparin as evident in our ThT assay, fluorescence imaging, STEM and DLS analysis (Fig. 4F–J). These results imply that PLGA can influence aggregation of both 3R as well as 4R tau, which are associated with the NFTs in AD brains^32^. In parallel, to evaluate if PLGA can influence tau aggregation induced by an Aβ seed other than Aβ_1-42_, we used Aβ_1-40_ known to be produced constitutively in normal brains^3,11,33^. Indeed, 2 µM Aβ_1-40_ seed, as observed with Aβ_1-42_, markedly enhanced aggregation kinetic of tau, which is attenuated by PLGA treatment. This is demonstrated using ThT kinetic assay, fluorescence imaging, STEM and DLS analysis (Fig. 5A–E; Supplementary Fig. 3E,F). Finally, to reveal if PLGA can influence tau aggregation by an inducer other than heparin, we measured the effects of 20 µM PLGA on 150 µM arachidonic acid-induced aggregation of 10 µM 0N4R tau with or without 2 µM Aβ_1-42_ seed. As observed with heparin, PLGA was able to potently suppress tau aggregation induced by arachidonic acid in the absence and presence of Aβ_1-42_ seed as apparent by ThT assay, fluorescence imaging, STEM and DLS analysis, suggesting that effect of native PLGA on tau aggregation does not depend on the presence of a specific inducer (Fig. 5F–J; Supplementary Fig. 3G,H).

### Specificity of PLGA effects on Aβ seed-induced tau aggregation

**Figure 6:**
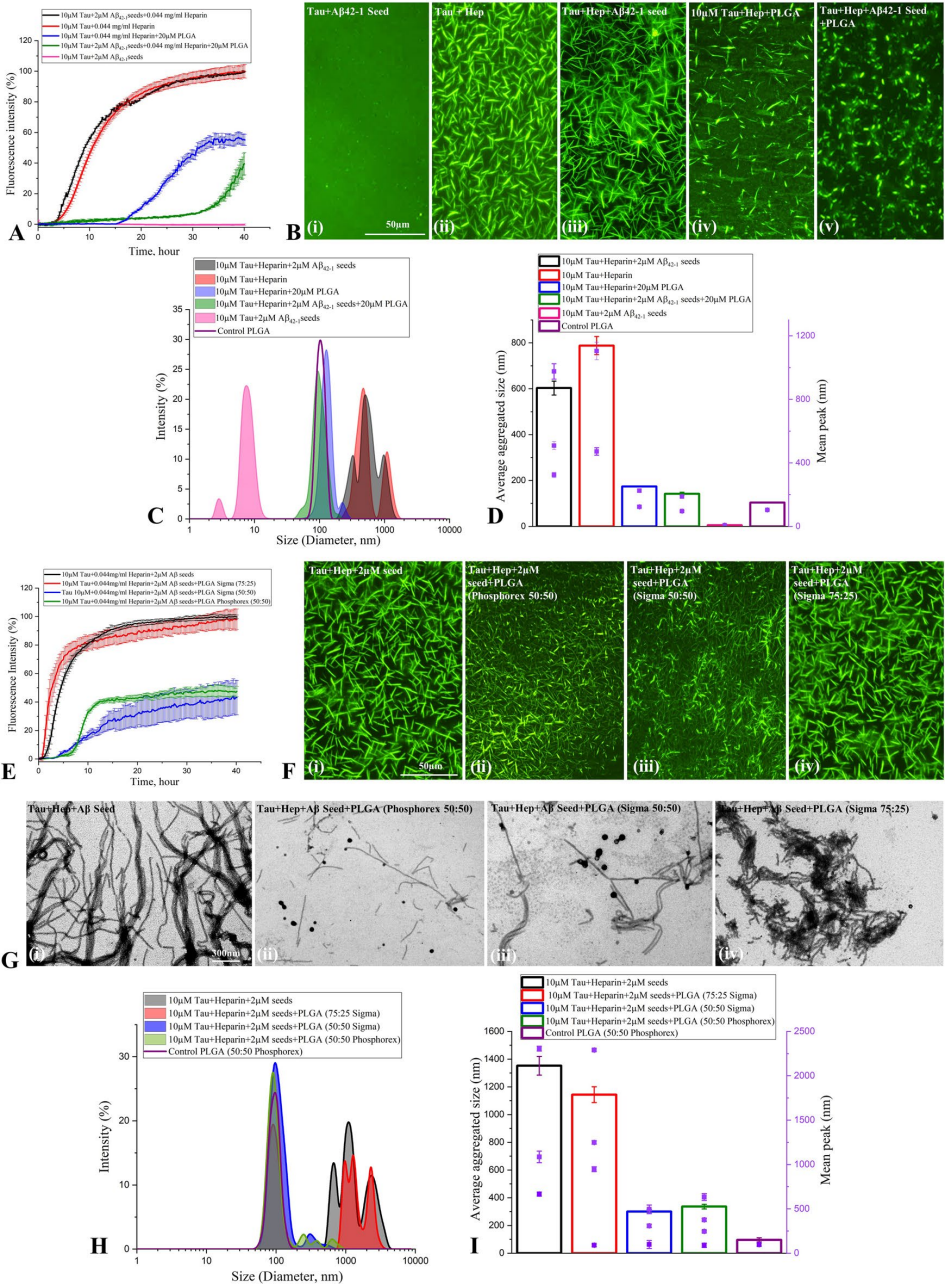
Specificity determining attenuation of tau aggregation by PLGA. (**A**,**B**) ThT kinetic assays (**A**) and fluorescence (**B**) images showing the effects 20 µM PLGA on Aβ_42-1_ seed-induced tau aggregation in the presence and absence of heparin following 40 h incubation. Note that Aβ_42-1_ seed did not trigger/alter aggregation of tau either in the absence or presence of heparin. PLGA suppressed tau aggregation observed in presence of heparin with or without Aβ_42-1_ seed. (**C**,**D**) DLS analysis of Aβ_42-1_ seed-induced tau aggregation revealed a differential decrease in the diameter of tau in the presence of 20 µM PLGA compared to control tau as depicted by size distribution curves (**C**) and and histogram (**D**) representing average aggregate size (left side Y axis) and mean peaks of tau aggregates for a given condition (right side Y axis). (**E**–**G**) ThT kinetic assays (**E**), fluorescence (**F**) as well as STEM (**G**) images showing that 50:50 resomer PLGA from Phosphorex and Sigma, but not 75:25 resomer PLGA, was able to suppress Aβ_1-42_ seed-induced tau aggregation over 40 h incubation. (**H**,**I**) DLS analysis showing size distribution curves (**H**) and and histogram (**I**) representing average aggregate size (left side Y axis) and mean peaks of tau aggregates for a given condition (right side Y axis) in the presence and absence of 20 µM PLGA of different resomers. Note the attenuation of tau aggregation by 50:50 resomer PLGA but not with 75:25 PLGA resomer compared to control. All ThT kinetic graphs represented average mean ± SEM of three separate experiments, each performed with six replicates for each condition.

First to establish the specificity tau aggregation induced by Aβ_1-42_ seed, we measured the effects of reverse Aβ_1-42_ (i.e., Aβ_42-1_—a negative control) on aggregation of 10 µM tau using ThT kinetic assay, fluorescence imaging and DLS. In contrast to normal sequence, 10 µM Aβ_42-1_ did not influence tau aggregation in the presence or absence of heparin, thus validating the specificity of the effect (Fig. 6A). As expected, PLGA attenuated heparin-induced tau aggregation with or without Aβ_42-1_ seed (Fig. 6A–D). To verify the specificity of PLGA's effect on tau kinetics, we demonstrated that PLGA with 50:50 resomer from Sigma-Aldrich can also suppress Aβ_1-42_ seed-induced tau aggregation, whereas equimolar PLGA with a 75:25 resomer composition did not affect Aβ_1-42_ seed-induced tau aggregation (Fig. 6E). These results, apart from ThT kinetic assays, were validated using fluorescence imaging, STEM images and DLS analysis, indicating that suppression of tau aggregation is primarily influenced by 50:50 resomer of PLGA (Fig. 6F–I) which is commonly used in various biological experiments^34^.

## Discussion

The present study using a variety of biophysical/structural studies revealed that biodegradable native PLGA nanoparticles, which have been clinically approved for drug-delivery^16,17^, can attenuate Aβ seed-induced aggregation of tau protein. This is supported by our data which show that: (i) Aβ_1-42_ seed dose-dependently increase tau aggregation with or without heparin and arachidonic acid, (ii) native PLGA nanoparticles attenuate Aβ seed-induced tau aggregation both in the presence and absence of heparin and arachidonic acid, (iii) native PLGA nanoparticles inhibit aggregation of both 4R and 3R tau which are present in NFTs and (iv) PLGA resomer of 50:50, but not 75:25, can able to decrease tau aggregation. Collectively, these results suggest that PLGA nanoparticles, without conjugation with any drug/agent, can decrease aggregation of tau protein which is critical in the development/spreading of tau pathology in AD-related disorders.

Under normal conditions, wild-type tau is a natively unfolded cytoplasmic protein involved primarily in the stabilization of microtubules without any propensity to aggregate^35^. Alterations in the MTBDs initiated by phosphorylation and/or other post-translational modifications such acetylation, methylation, glycation and ubiquitination trigger conversion of monomeric tau into a pathological aggregated form. The first step in the conversion involves tau dimerization occurring either by intermolecular disulfide crosslinking between two cystine residues located in the R2 (Cys291) and R3 (Cys322) domains or by electrostatic interactions between the positively charged proline-rich domain and the negatively charged N-terminal domain and MTBD. The tau dimers then self-assemble via aggregate-prone hexapeptide motifs (i.e. VQIVYK and VQIINK) on MTBDs to form oligomers, which grow into fibrils composed of steric ‘zippers’ of two tightly integrated β-sheet structures leading to the formation of PHFs—the building blocks of NFTs^36^. Tau aggregation not only destabilizes cytoskeleton architecture leading to degeneration of neurons but also involve in the “seed-induced” spreading of disease pathology via prion-like mechanism from an affected region to an anatomically connected unaffected brain region^9,37,38^. The full-length recombinant wild-type tau, as observed in the present study, does not aggregate spontaneously due to its hydrophilic nature and/or lack of post-translational modifications^35^. Thus, tau aggregation under in vitro condition is usually studied by the addition of polyanionic factors such as heparin or arachidonic acid^39^. These cofactors, as demonstrated in our data, trigger tau aggregation by inducing a conformational change in the hexapeptide motifs in MTBDs – providing a platform to determine potential inhibitors of tau fibrillization^14,40^.

Multiple lines of evidence suggest that increased Aβ levels/accumulation can cause downstream signaling changes that can trigger phosphorylation and aggregation/seeding of tau protein leading to development of AD pathology^14,41,42^. This is supported by data showing that (i) Aβ aggregates appear prior to tau pathology in AD brains^2,3^, (ii) Aβ can induce tau phosphorylation in cultured neurons and in vivo conditions^43,44^, (iii) Aβ aggregates can trigger and/or propagate tau pathology in cultured cells and in a variety of animal models^45–48^. Pre-aggregated Aβ, as apparent in the present study, has also been shown to dose-dependently accelerate aggregation of tau protein under in vitro conditions^12,49^. Additionally, tau isolated from AD patients containing neuritic plaques exhibit enhanced ability to induce tau aggregation compared to tau isolated without plaques^13^. Similar results were obtained in transgenic mice overexpressing both mutated APP and tau^50^. It has been suggested that pre-aggregated Aβ can serve as template for tau aggregation by circumventing the required nucleation phase^12^. Although the underlying sequence-structure-aggregation relationship remains unclear, it is possible that Aβ seeds with stackable β-strands increase the hydrophobic surface area facilitating the template-assisted growth of tau protein^42^. In fact, it has been reported that Aβ core region along with its C-terminal residues can interact with the aggregation prone “VQIINK” and “VQIVYK” regions of tau to accelerate cross-seeding mediated aggregation of tau protein^14^. This is partly supported by detection of complexes containing Aβ bound tau in AD brain extracts^51^. It is of interest to note that both Aβ_1-42_ and Aβ_1-40_ seeds can enhance tau aggregation in the presence and absence of heparin/arachidonic acid suggesting that interaction sites underlying tau aggregation induced by Aβ and polyanionic factors are possibly complementary rather than overlapping.

Although Aβ dysfunction presages tau pathology, it is now believed that synergistic/cooperative interplay between Aβ and tau may underlie the development and spreading of AD pathology^3,52,53^. Thus, molecules that target different facets of both Aβ and tau pathology may offer a better prospect to treat AD than those targeting either protein independently. Recently we have reported that native PLGA can inhibit not only spontaneous aggregation of Aβ but also can trigger dismantling of Aβ aggregates in a dose-dependent manner^28,29^. The present results using ThT kinetic assay showed that Aβ seed-induced tau aggregation with or without heparin is inhibited by native PLGA. These data are validated by fluorescence imaging and STEM depicting the presence of shorter and fragmented fibrils/globular aggregates as well as by DLS analyses in which large fibrils that normally dominate the light scattering are found to be reduced to smaller species. The inhibition, which is apparent as a function of PLGA concentration, is also noted in the presence of arachidonic acid suggesting that the effect is independent of tau aggregation inducers. It is of interest to note that heparin-/arachidonic acid-induced tau aggregation was attenuated in the absence of Aβ seeds. The inhibitory effect of PLGA is facilitated not only by delaying the lag phase but also decreasing the growth and saturation of tau fibrils during the linear and equilibrium phases, respectively – indicating that all stages of tau fibrillogenesis are likely affected. This could be due to the interaction of PLGA with Aβ seed precluding the hydrophobic contacts required for the template-assisted growth of tau protein leading to fibril formation. It is also possible that PLGA by interacting with Aβ core region can attenuate the interaction of Aβ with the aggregation prone “VQIINK” and “VQIVYK” regions of tau resulting in the destabilization of aggregation kinetics and formation of tau intermediates containing a mixture of α-helical and β-sheet conformations. Additionally, the evidence that native PLGA can suppress tau aggregation in the absence of Aβ seed, it could not be excluded that PLGA by interacting directly with the aggregate-prone hexapeptide motifs on MTBDs can hinder tau dimerization and/or its assembly. Furthermore, as native PLGA nanoparticles are able to suppress tau fibrilization irrespective of Aβ seed and also can attenuate Aβ aggregation by interacting with its hydrophobic domain Lys_16_ to Ala_21_^28^, it is of interest to determine if PLGA prevents tau aggregation by intervening with the common interface regulating tau and Aβ binding. Our results further revealed that native PLGA inhibits aggregation of both 4R tau as well as 3R tau – which exhibit different aggregation kinetics but are found together in the NFTs^32^. The suppressing effects of PLGA on tau aggregation is evident in the presence of both Aβ_1-42_ and Aβ_1-40_ thus suggesting that native PLGA regardless of Aβ and tau isoforms can suppress aggregation of tau protein. The specificity of the effects is established by demonstrating that normal Aβ_1-42_ and Aβ_1-40_, but not Aβ_42-1_ sequence, were able to accelerate aggregation of tau protein in the presence of heparin. Additionally, our result from ThT kinetic assays revealed that native PLGA resomer with a lactide-glycoside ratio of 50:50 from two different sources, but not 75:25 resomer, can suppress tau fibrillization as observed with inhibition of spontaneous Aβ aggregation under in vitro conditions^29^. At present, the precise mechanisms underlying the differential effects of PLGA 50:50 *vs.* PLGA 75:25 on tau aggregation remain unclear. Evidence suggests that interaction of PLGA polymers with biologically active molecules depends on their surface properties. PLGA 50:50, with its balanced ratio of lactic to glycolic acids presents a more hydrophilic surface^54,55^ than the more hydrophobic PLGA 75:25 resomer. Tau protein, being predominantly hydrophilic and enriched with charged amino acids, it is likely to interact favorably with hydrophilic surfaces of PLGA 50:50 which could sterically hinder self-association of tau molecules leading to reduction in its aggregation. The faster degradation kinetics of PLGA 50:50 due to its higher glycolic acid content compared to PLGA 75:25 may also play a role in its interaction with tau protein. Since tau is positively charged, particularly in its microtubule-binding domain, the increased negative charge density on the surface of PLGA 50:50 may foster stronger electrostatic interactions with the positive charges on tau protein or sterically block its aggregation-prone regions, which could reduce its propensity to aggregate. Lastly, considering the evidence that native PLGA that has been reported to inhibit Aβ aggregation^28,29^ can suppress tau fibrilization raise the possibility of a common aggregation interface between tau and Aβ peptides.

Despite extensive research, at present there is no effective treatment to arrest the advancement of AD pathology. The acetylcholinesterase inhibitors (Donepezil, Galantamine and Rivastigmine) and the glutamatergic NMDA receptor antagonist (i.e. Memantine) that have been approved by FDA for the treatment provide symptomatic relief for only a fraction of AD patients^33,56^. The efficacy of newly approved Aβ monoclonal antibodies Aducanumab and Lecanemab on AD patients remain to be determined^57^. Earlier studies have shown that PLGA nanoparticles when conjugated/encapsulated with curcumin, methylene blue or a mixture of rosmarinic acid/curcumin can able to attenuate tau aggregation and exhibit beneficial effects on tau pathology in cellular and/or animal models of AD compared to drugs alone or vehicles used for dissolving drugs^26,58,59^. However, no study has been carried out so far to determine if native PLGA can influence tau aggregation or the development/spreading of tau pathology in an animal model of AD. As a follow up to a report on cellular/animal models of Parkinson disease^60^, we showed that native PLGA can attenuate aggregation of Aβ peptide and exhibit beneficial effects in the 5xFAD mouse model of AD. Additionally, our results revealed that native PLGA can protect cultured mouse neurons as well as induced pluripotent stem cell-derived AD neurons against Aβ-mediated toxicity by reducing tau phosphorylation^27–29,61^—which plays a critical role in triggering aggregation and formation of NFTs in AD brains. These results, together with the evidence that PLGA can attenuate Aβ seed-induced tau aggregation, not only suggest a role for native PLGA on different facets of tau protein but also highlight its unique potential to treat AD by targeting simultaneously both Aβ and tau pathology.

## Methods

### Materials

Recombinant human Aβ_1-42_ (> 97% purity) and Aβ_1-40_ (> 95% purity) were purchased from rPeptide R&D (Sunnyvale, CA, USA). Human Aβ_42-1_ (> 95% purity) was purchased from Anaspec (Sunnyvale, CA, USA), whereas PLGA (50:50 resomer; lactic acid:glycolic acid; mol. wt. ~ 30,000) was from Phosphorex (Hopkinton, MA, USA). Hexafluoro-2-Propanol (HFIP), ThT, Dithiothreitol (DTT), dimethyl sulfoxide (DMSO), PLGA (50:50 and 75:25 resomers; lactic acid:glycolic acid) were procured from Sigma-Aldrich (St. Louis Missouri, USA). The tau aggregation accelerants heparin sodium salt and arachidonic acid were purchased from Santa Cruz and Sigma-Aldrich, respectively. Electron microscopy grids (carbon coated 400 mesh copper grids) and uranyl acid stains were obtained from Electron Microscopy Sciences (PA, USA). All other standard chemicals were from either Sigma-Aldrich or Thermo Fisher Scientific.

### Preparation and purification of human recombinant tau

Tau was expressed and purified as previously described^62^. In brief, 0N4R and 1N3R tau expression in *E.coli* was induced in media (terrific broth, 10 mM betaine, 500 mM NaCl) with 500 μM isopropyl-thio-β-d-galactoside for 3 h at 30 °C. Following expression, cells were lysed via a microfluidizer and the cell lysate was boiled. The clarified supernatant containing soluble tau was subject to cation exchange chromatography (20 mM MES pH 6.8, 2 mM DTT, 1 mM MgCl_2_, 1 mM EGTA, 50–600 mM NaCl). Purified tau fractions were pooled and dialyzed into aggregation assay buffer [Dulbecco’s phosphate buffered saline (PBS, pH 7.4) 2 mM DTT] then concentrated with a 3 kDa MWCO filter. Protein concentration was determined with a Pierce™ BCA Protein Assay Kit—Reducing Agent Compatible. Purified protein aliquots are stored at − 80 °C prior to use.

### Preparation of Aβ peptides

Lyophilized Aβ_1-42,_ Aβ_1-40_ and Aβ_42-1_ were first equilibrated at room temperature for 30 min and then dissolved in HFIP to obtain a 1 mM solution. Once dissolved, peptide aliquots were allowed to dry using a SpeedVac to remove HFIP and then stored at − 80 °C for subsequent analysis^29^. On the day of experiment, Aβ_1-42,_ Aβ_1-40_ and Aβ_42-1_ aliquots were thawed at 4 °C, diluted with DMSO to obtain 5 mM concentration and then further diluted to 100 μM with PBS and subsequently incubated at 37 °C for 24 h to generate Aβ fibrils. Aβ seeds were prepared by sonicating preformed Aβ fibrils using a probe sonicator with 40 pulses and 30% amplitude.

### Preparation of PLGA

PLGA nanoparticles were prepared following manufacturer’s instruction as described earlier^28,29^. In brief, PLGA powder was dissolved into 0.01 M PBS (pH 7.4) followed by sonication using a probe sonicator with 40 pulses and 40% amplitude.

### In vitro tau aggregation

The aggregation kinetic assays for wild-type 0N4R tau (2.5–20 μM) and 1N3R tau (10 µM) were carried out in 150 μL assay buffer (Dulbecco’s PBS, 2 mM MgCl_2_, 1 mM DTT, pH 7.2) at 37 °C with heparin sodium salt (0.044 mg/ml final concentration) in the absence or presence of different concentrations (1 µM, 2 µM or 5 µM) of Aβ_1-42_ seeds or 2 µM Aβ_1-40_ seeds as described earlier^29,63^. In the case of uninduced controls, assay buffer was added in place of heparin solution. Subsequently, aggregation of 10 μM 0N4R tau and 1N3R tau with or without heparin + 2 µM Aβ_1-42_-seed was evaluated in the absence or presence of various concentrations (1 µM, 5 μM, 10 μM, 15 µM and 20 μM) native PLGA. In parallel, we determined the effects of 20 µM PLGA on the aggregation kinetics of 0N4R tau (10 μM) induced by 150 μM arachidonic acid in the absence or presence of 2 µM Aβ_1-42_ seed. In some experiments, 0N4R tau (10 μM) aggregation kinetics were determined in presence of 20 µM PLGA (50:50 and 75:25 resomers) obtained from Sigma-Aldrich. Aggregation of 10 μM 0N4R tau was also studied with 2 μM Aβ_42-1_ seed with or without native PLGA as a control. All aggregation processes were performed by monitoring the fluorescence of ThT (20 μM) present in reactions. The fluorescence signal was measured every 15 min for 40 h using a FLUOstar omega BMG Labtech (Aylesbury, UK) with excitation at 440 nm, emission at 480 nm and a 475 nm emission cutoff. The lag time of tau aggregation observed with various conditions was determined by selecting a common fraction of fluorescence signal intensity (i.e. 5%) relative to pre-transition base line as described earlier^29^. All kinetic experiments were repeated at least three times with six technical replicates for each sample/experiment and the final data are presented as mean + SEM. Raw data from various experiments were normalized as % fluorescence intensity considering highest fluorescence intensity value obtained in each experiment as 100% and then graphs were generated using ORIGIN 2018.

### Fluorescence microscopy

A small aliquot (10 μL) of 0N4R and 1N3R tau samples from various experimental conditions in the absence and presence of heparin, arachidonic acid, Aβ (Aβ_1-42_, Aβ_1-40_ and Aβ_42-1_) seeds and PLGA were added on a clean glass slide, air-dried and then stained with ThT solution as described earlier^29^. The images of ThT-stained 0N4R and 1N3R tau aggregates were then photographed using a fluorescence microscope (Nikon eclipse 90i) at 20X magnification.

### Scanning transmission electron microscopy (STEM)

The morphological changes of 0N4R and 1N3R tau samples obtained from various experimental paradigms were evaluated using an ultra-high resolution Hitachi S-5500 cold field emission STEM. Initially 5 µL tau samples with or without Aβ seeds and PLGA were deposited onto plasma cleaned, carbon coated copper grids for 30 s, blotted to remove excess liquid, air-dried and then softly washed with 10 µL of Milli-Q water to remove the salt. The grids were subsequently stained with 2% aqueous uranyl acetate for 30 s, blotted to take off excess liquid, dried and then imaged at 30 kV accelerating voltage and 30 µA emission current as described previously^29^.

### Dynamic light scattering (DLS)

The size distribution of various aggregated tau samples with or without PLGA + Aβ seeds were carried out at the end of the saturated phase (i.e. 40 h) using a Malvern Zetasizer-Nano Instrument as described earlier^29^. In a given experiment the size distribution of tau aggregates with or without PLGA was monitored at different time points (0 h, 4 h, 20 h and 40 h) during the aggregation process. A He–Ne laser with a wavelength of 632 nm was used to detect backscattered light at a fixed angle of 173°. The aggregated tau samples were prepared by incubating 10 μM tau with or without heparin, 1–5 µM Aβ seeds and 5–20 μM PLGA at 37 °C under constant shaking. The software (DTS v6.20) provides both the mean size and polydispersity by cumulants analysis. For calculation purposes, the solution viscosity and refractive index (1.33) were assumed to be of water. Data were collected from a minimum number of 10 consecutive runs of 10 s each using a 10 mm quartz cuvette filled with 150 μL sample without agitation to obtain the autocorrelation function. The size of the particle was calculated by the manufacturer’s software through the Stokes–Einstein equation.

### Circular dichroism (CD)

CD experiments were performed using a Chirascan circular dichroism spectrometer (Applied photophysics) as described earlier^29^. The CD spectra of_._ aggregated Aβ seed-induced tau with or without heparin and 20 µM PLGA (at 1:2 ratio) at 37 °C were recorded over a wavelength range of 250–190 nm, by using 0.1 cm path length quartz cell. Each spectrum was averaged using 6 repeat scans. The CD spectra of all samples were measured at 40 h (i.e. after saturation) to evaluate the effect of PLGA on the aggregation process. Baseline correction was carried out by subtracting the spectral contribution of an assay buffer containing Dulbecco’s PBS, 2 mM MgCl_2_ and 1 mM DTT. To obtain a better quantitative structural information, all raw CD spectra were de-convoluted using the CDPro software.

### Supplementary Information

Supplementary Figures.

## Data Availability

The datasets used and/or analyzed during the current study available from the corresponding author on reasonable request.

## Abbreviations

Aβ: Amyloid β
AD: Alzheimer’s disease
APP: Amyloid precursor protein
BBB: Blood brain-barrier
CD: Circular dichroism
DLS: Dynamic light scattering
DMSO: Dimethyl sulfoxide
DTT: Dithiothreitol
HFIP: Hexafluoro-2-propanol
MTBD: Microtubule binding domain
NFTs: Neurofibrillary tangles
PBS: Phosphate-buffered saline
PHFs: Paired helical filaments
PLGA: Poly(D,L-lactide-co-glycolide)
STEM: Scanning transmission electron microscopy
ThT: Thioflavin-T
