# Supplementary material for: Native PLGA nanoparticles attenuate Aβ-seed induced tau aggregation under in vitro conditions: potential implication in Alzheimer’s disease pathology
Source: Sci Rep. 2024 Jan 2;14:144. doi: 10.1038/s41598-023-50465-x (PMC10762165; doi:10.1038/s41598-023-50465-x)

Legends of Supplementary Figures

Supplementary Figure 1

A and B; Histograms showing the lag phase (A) and the overall amount at saturation (B) following dose-dependent aggregation of tau in the absence and presence of heparin as detected using ThT kinetic assay over 40 hr incubation. **C and D**; DLS analysis showing an increase in the diameter of aggregated tau as a function of time in the presence of heparin over 40 hr incubation as depicted by size distribution curves (C) and and histogram (D) representing mean peaks of tau aggregates at a given time. **E and F**; ThT kinetic assays showing fluorescence intensity observed over 40 hr period of spontaneous aggregation of 1-5 µM Aβ_{1-42} seeds in the presence of heparin with (E) or without (F) 10 µM 0N4R tau. Note the dose-dependent increase in the aggregation profile of 1, 2 and 5 µM Aβ_{1-42} in the presence of heparin but the fluorescence intensity values are quite low compared to those observed in presence of 10 µM 0N4R tau. **G and H**; Histograms showing the lag phase (G) and the overall amount at saturation (H) following aggregation of 10 µM 0N4R tau in the presence of heparin with or without 1-5 µM Aβ_{1-42} seeds as detected using ThT kinetic assay over 40 hr incubation. **I and J**; Histograms showing the lag phase (I) and the overall amount at saturation (J) following aggregation of 10 µM 0N4R tau in the absence of heparin with 1-5 µM Aβ_{1-42} seeds as detected using ThT kinetic assay over 40 hr incubation.

Supplementary Figure 2

A; ThT kinetic assays showing the real value of fluorescence intensity of 10 µM tau, 10 µM tau+0.044 mg/ml heparin, 10 µM tau+0.044 mg/ml heparin+5 µM Aβ_{1-42} seed, 10 µM Tau+5 µM Aβ_{1-42} seeds and 5 µM Aβ_{1-42} seed only measured at 482 nm. **B**, DLS histogram of the PLGA nanoparticles displaying the diameter size of ~100 nm. **C**, STEM images showing the spheroidal nature of PLGA nanoparticles with an average diameter of ~100 nm. **D** and **E**, Histograms showing the lag phase (D) and the overall amount at saturation (E) following Aβ_{1-42} seed-induced spontaneous aggregation of 10 µM 0N4R tau in the presence of heparin and PLGA (1-20 µM) as detected using ThT kinetic assay over 40 hr incubation.

Supplementary Figure 3

A and B; Histograms showing the lag phase (A) and the overall amount at saturation (B) following spontaneous aggregation of 10 µM 0N4R tau without heparin but in the presence of Aβ_{1-42} seeds and PLGA as detected using ThT kinetic assay over 40 hr incubation. **C and D**; Histograms showing the lag phase (C) and the overall amount at saturation (D) following aggregation of 10 µM 0N4R tau in the presence of heparin and PLGA without Aβ_{1-42} seeds as detected using ThT kinteic assay over 40 hr incubation. **E and F**; Histograms showing the lag phase (E) and the overall amount at saturation (F) following Aβ_{1-40} seed-induced aggregation of 10 µM 0N4R tau with or without heparin and PLGA as detected using ThT kinteic assay over 40 hr incubation. **G and H**; Histograms showing the lag phase (G) and the overall amount at saturation (H) following Aβ_{1-42} seed-induced aggregation of 10 µM 0N4R tau in the presence of arachidonic acid and PLGA as detected using ThT kinetic assay over 40 hr incubation.

Supplementary Figure 1

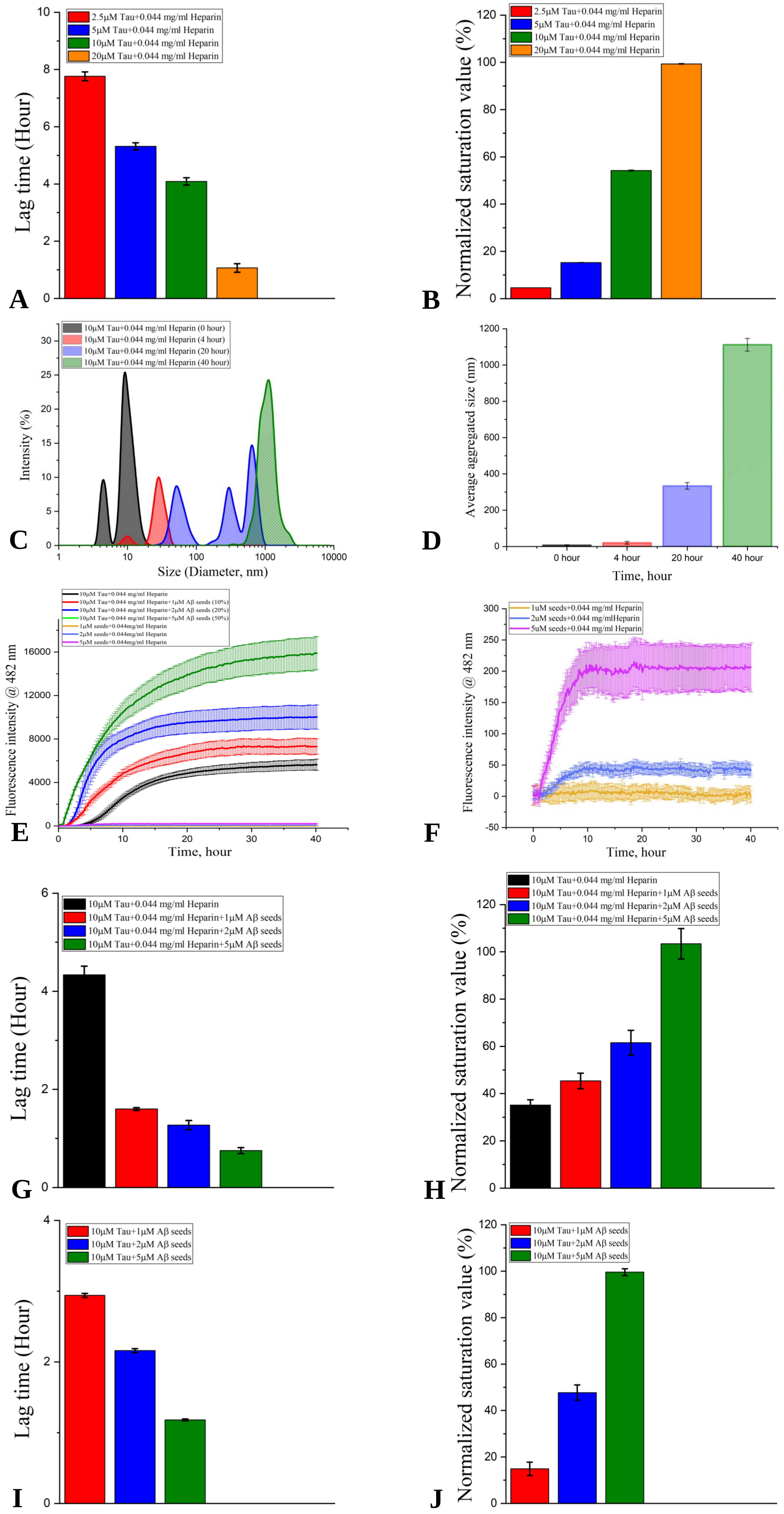

Supplementary Figure 2

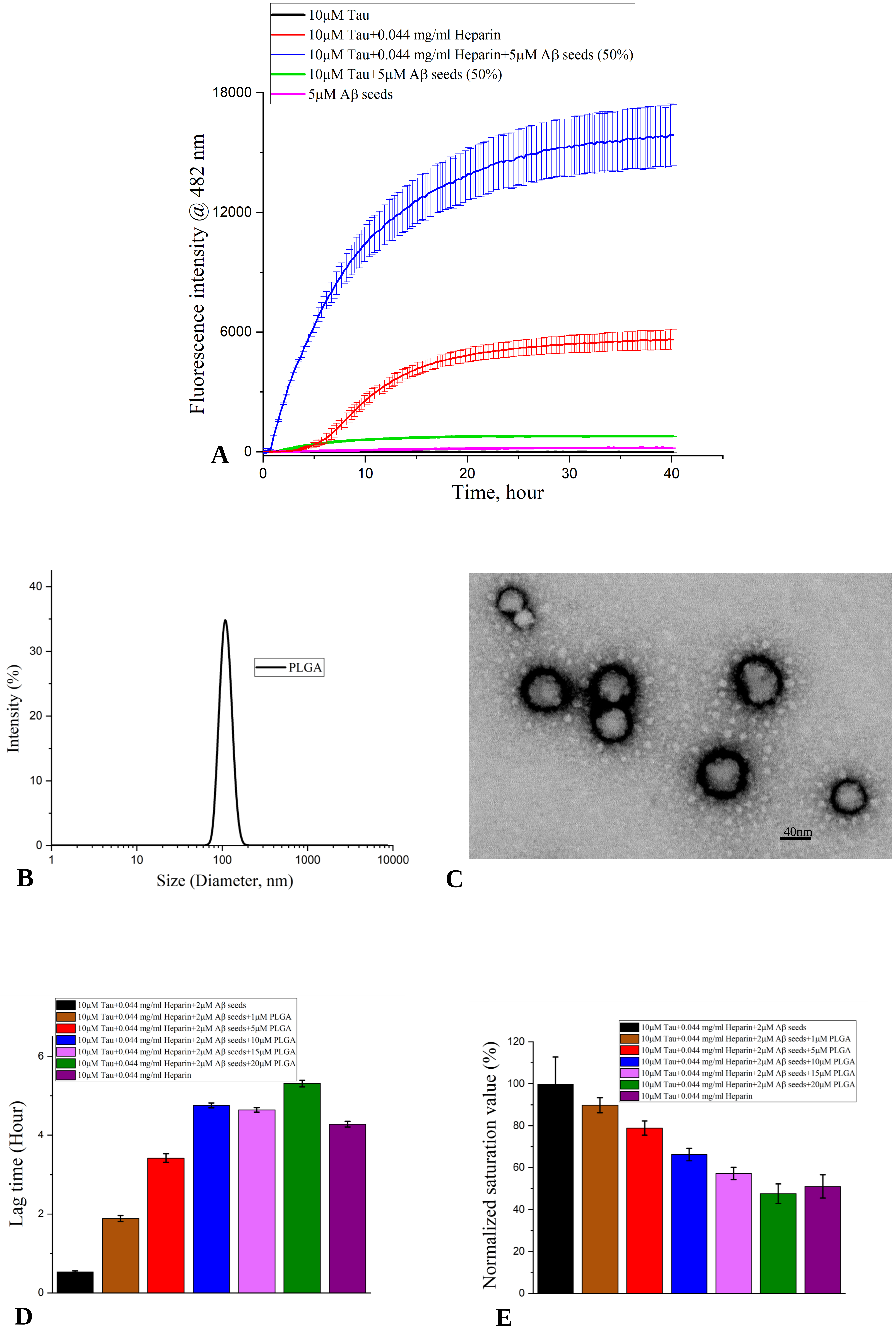

Supplementary Figure 3

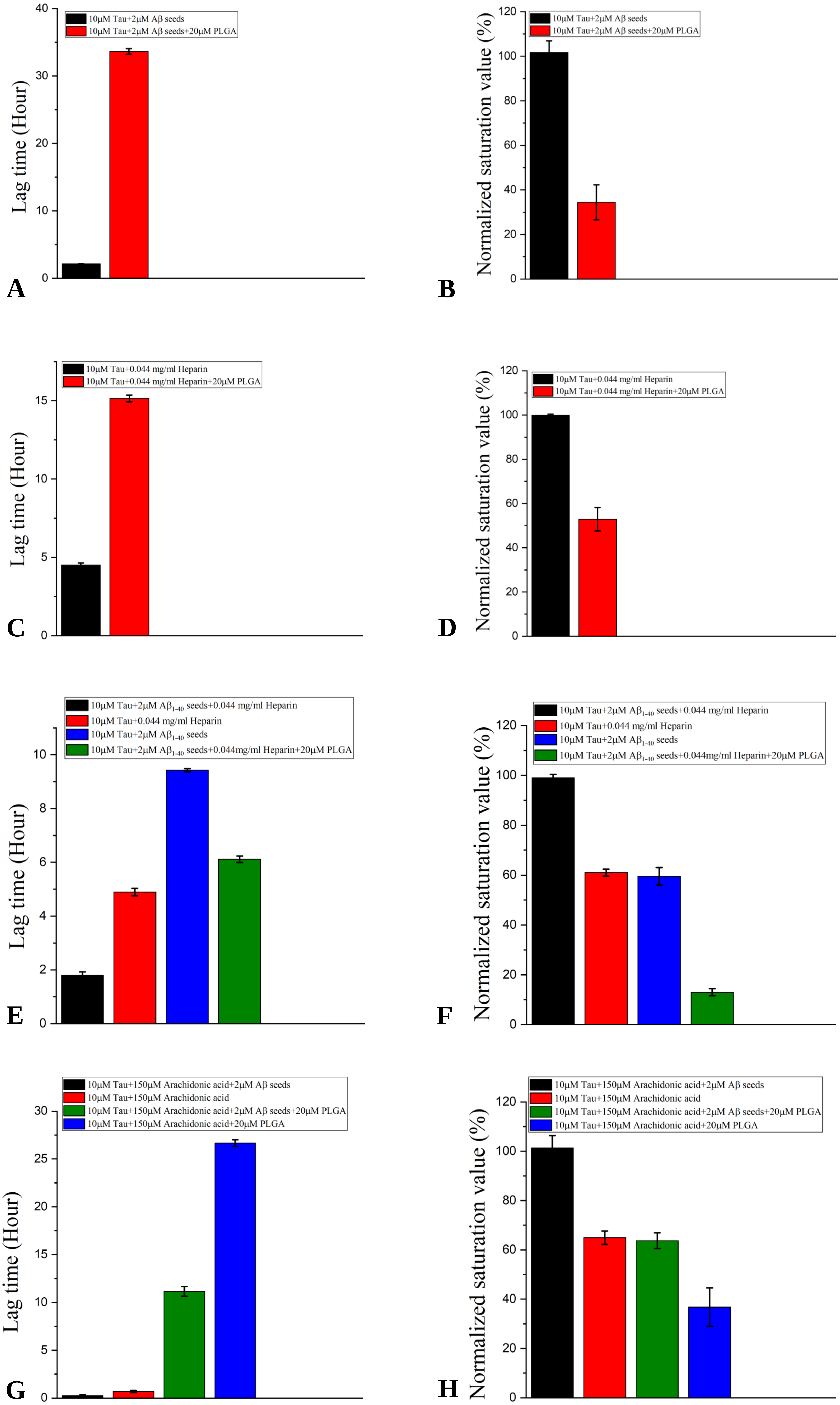

Supplement: Supplementary file 1 — Supplementary Figures. [file 41598_2023_50465_MOESM1_ESM.pdf]
